# Hyperglycemia-related advanced glycation end-products is associated with the altered phosphatidylcholine metabolism in osteoarthritis patients with diabetes

**DOI:** 10.1371/journal.pone.0184105

**Published:** 2017-09-12

**Authors:** Weidong Zhang, Edward W. Randell, Guang Sun, Sergei Likhodii, Ming Liu, Andrew Furey, Guangju Zhai

**Affiliations:** 1 Discipline of Genetics, Faculty of Medicine, Memorial University of Newfoundland, St John’s, Newfoundland and Labrador, Canada; 2 School of Pharmaceutical Sciences, Jilin University, Changchun, P.R. China; 3 Department of Laboratory Medicine, Faculty of Medicine, Memorial University of Newfoundland, St John’s, Newfoundland and Labrador, Canada; 4 Discipline of Medicine, Faculty of Medicine, Memorial University of Newfoundland, St John’s, Newfoundland and Labrador, Canada; 5 Department of Surgery, Faculty of Medicine, Memorial University of Newfoundland, St John’s, Newfoundland and Labrador, Canada; 6 Menzies Research Institute, University of Tasmania, Hobart, Tasmania, Australia; Augusta University, UNITED STATES

## Abstract

To test whether type 2 diabetic patients have an elevated level of advanced glycation end-products (AGEs) and responsible for altered phosphatidylcholine metabolism, which we recently found to be associated with osteoarthritis (OA) and diabetes mellitus (DM), synovial fluid (SF) and plasma samples were collected from OA patients with and without DM. Hyperglycemia-related AGEs including methylglyoxal (MG), free methylglyoxal-derived hydroimidazolone (MG-H1), and protein bound N-(Carboxymethyl)lysine (CML) and N-(Carboxyethyl)lysine (CEL) levels were measured in both SF and plasma samples using liquid chromatography coupled tandem mass spectrometry methodology. The correlation between these AGEs and phosphatidylcholine acyl-alkyl C34:3 (PC ae C34:3) and C36:3 (PC ae C36:3) were examined. Eighty four patients with knee OA, including 46 with DM and 38 without DM, were included in the study. There was no significant difference in plasma levels of MG, MG-H1, CML, and CEL between OA patients with and without DM. However, the levels of MG and MG-H1, but not CML and CEL in SF were significantly higher in OA patients with DM than in those without (all p ≤0.04). This association strengthened after adjustment for age, body mass index (BMI), sex and hexose level (p<0.02). Moreover, the levels of MG-H1 in SF was negatively and significantly correlated with PC ae C34:3 (ρ = -0.34; p = 0.02) and PC ae C36:3 (ρ = -0.39; P = 0.03) after the adjustment of age, BMI, sex and hexose level. Our data indicated that the production of non-protein bound AGEs was increased within the OA-affected joint of DM patients. This is associated with changes in phosphatidylcholine metabolism and might be responsible for the observed epidemiological association between OA and DM.

## Introduction

Osteoarthritis (OA) is the most common joint disease worldwide, affecting 10% of men and 18% of women over 60 years of age [1]. Accumulating evidence suggests that OA is associated with metabolic syndrome (MetS) related conditions, especially type 2 diabetes mellitus (DM) [2–6]. While the potential mechanisms for the association between OA and DM remain unclear, we [7] recently found that phosphatidylcholine acyl-alkyl C34:3 (PC ae C34:3) and C36:3 (PC ae C36:3) were associated with the concurrence of OA and DM, suggesting that altered phosphatidylcholine metabolism might be responsible for the observed association between OA and DM.

There is substantial evidence to suggest that intracellular glucose toxicity in DM may be mediated through increased production of highly reactive α-ketoaldehydes [8–10]. The non-enzymatic reaction of α-ketoaldehydes with protein produces advanced glycation end-products (AGEs). The accumulation of AGEs may play a causal role in the development of complications of DM including OA. AGEs have also been implicated in OA [11,12].

AGEs can modify normal macromolecules functions directly or by generating reactive oxygen species (ROS) either independently, or by activation of the receptor for AGEs (RAGE) [13–15]. It has been reported that ROS can result in membrane lipid peroxidation [16]. Lipid peroxidation is the process in which free radicals "steal" electrons from the lipids in cell membranes, resulting in cell damage. This process proceeds by a free radical chain reaction mechanism. It often affects polyunsaturated fatty acids, because they contain multiple double bonds in between which lie methylene bridges (-CH2-) that possess especially reactive hydrogen atoms. PC ae C34:3 and PC ae C36:3 belong to a special but sub-group of unsaturated phosphatidylcholines which comprise an ether linkage to one alkyl chain and one polyunsaturated fatty acid and have unique bioactivity, including a role in generation of lipid second messengers, but also providing a protective effect against oxidative stress [17]. The decreased concentrations of both unsaturated PC metabolites in OA with DM may be related to an increased potential for lipid peroxidation with AGE potentially playing a pathogenic role. We therefore undertook this study to examine whether plasma and synovial levels of AGEs were increased in OA patients with DM, and determined how this might be related to the levels of PC ae C34:3 and PC ae C36:3.

## Patients and methods

### Patients

The study was part of the ongoing Newfoundland Osteoarthritis Study (NFOAS) [18]. Patients with OA were recruited from those who underwent total knee replacement surgery due to primary OA between November 2011 and December 2014 in St. Clare’s Mercy Hospital and Health Science Centre General Hospital in St. John’s, the capital of Newfoundland and Labrador, Canada. Demographic and medical information was collected by a self-administered general questionnaire with the assistance of research staff if necessary. DM status of the patients were determined by the self-reported general questionnaire and confirmed by their hospital diagnosis records. The study was approved by the Health Research Ethics Authority of Newfoundland and Labrador (reference number is 11.311) and written consent was obtained from all the participants.

### Measurement of PC ae C34:3 and PC ae C36:3 in plasma and synovial fluid (SF)

Blood samples were collected after overnight fast of at least 8 h, and SF samples were collected during joint replacement surgeries. Plasma and SF samples were processed as previous described [7]. Metabolic profiling in both plasma and SF were performed by the Waters XEVO TQ MS system (Waters Ltd.) using the Biocrates AbsoluteIDQ p180 kit as part of a previous project [18]. Data on PC ae C34:3 and PC ae C36:3 were retrieved from the metabolic profile data for the purposes of the current study.

### Measurement of methylglyoxal (MG) and hydroimidazolone (MG-H1) in plasma and SF

MG was measured by liquid chromatography coupled with tandem mass spectrometry (LC-MS/MS) using methodology previously developed by our group [19]. Briefly, small aliquots of samples (~100 μL; SF and plasma) was derivatized using 2, 3-diaminonaphthalene (2, 3-DAN) and extracted, and dried under nitrogen gas. The dried extracts were reconstituted with acetonitrile for LC-MS/MS analysis. The 2, 3-DAN derivative of methylglyoxal was separated in an isocratic solvent system consisting of 0.1% formic acid and acetonitrile (35/65, v/v) using a C8 column (3.5 μm, 2.1×100 mm, Waters, Massachusetts, USA) at a flow rate of 0.30 mL/min at a temperature of 25°C. The derivative of methylglyoxal was determined by selective reaction monitoring of the transition m/z 195>168 and using 5-methylquinoline (m/z 145>91) as an internal standard.

MG-H1 was measured by LC-MS/MS using methodology previously developed in our laboratory [20]. Briefly, samples (synovial and plasma) will be extracted by strong anion exchange solid phase extraction columns (Oasis MCX 1cc 30 mg, Waters, Massachusetts, USA). The dried extracts containing MG-H1 was reconstituted with methanol for LC-MS/MS by the Water Alliance HT 2795-Micromass Quattro Ultima LC-MS/MS system. MG-H1 was separated in a gradient solvent system consisting of 10 mM formic acid in methanol, water, and acetonitrile at a flow rate of 0.6 mL/min at ambient temperature on a Hilic Sillica column (Atlantis^®^ Hilic Slica, 3 μm, 2.1×50 mm, Waters Corporation, Massachusetts, USA).

### Measurement of N-(Carboxymethyl)lysine (CML) and N-(Carboxyethyl)lysine (CEL) in plasma and SF

CML and CEL were measured by LC-MS/MS using methodology previously developed by Teerlink et al. [21] but with minor modifications to the gradient system to optimize separation using our system. Briefly, sample were analyzed at ambient temperature by reversed-phase HPLC on a Symmetry C18 column (5μm, 3.9×150mm, Waters, Massachusetts, USA) and using nonafluoropentanoic acid (NFPA) as the ion pairing agent in a two solvent gradient separation involving acetonitrile as the organic phase. Analyses were performed in positive-ion mode but on the Water Alliance HT 2795-Micromass Quattro Ultima LC-MS/MS system. For both CML and CEL, the product ion at m/z 84.1 was used for quantification and the product ion at m/z 130.1 for confirmation. In each series, five calibration samples spanning the concentration ranges of 0–2.5 μmol/L for CML and 0–1.0 μmol/L for CEL were included.

### Statistical methods

The distribution of the concentrations of PC ae C34:3, PC ae C36:3, MG, MG-H1, CML, and CEL in plasma and SF samples were examined by qq plot and transformed by log_10_ transformation where necessary. The missing values (no data) were replaced by half of the minimum value found in the dataset [22]. The comparisons of the four AGEs between OA with and without DM were tested by *t*- test, and visualized by boxplots. The correlation between each of the four AGEs and PC ae C34:3 and PC ae C36:3 were examined by Spearman correlation coefficient. Statistically significant results were identified by p≤0.05. The potential confounders including age, sex, body mass index (BMI) and hexose level (>90% is glucose) were adjusted by linear regression modelling. All the statistical analysis was carried out using PCIT package and ggplot2 Graphics packages implemented in R (version 3.1.1 for Windows).

## Results

In total, 84 knee OA patients, including 46 with DM and 38 without DM, were included in the study. Basic descriptive demographics of the study population are presented in Table 1. The number of males and females in the DM and non-DM groups were similar, but OA patients with DM were older and had a higher BMI than OA patients without DM (Table 1; all p≤0.01). We measured the total hexose levels in both plasma and SF samples, which is mainly represented by glucose. The plasma and SF hexose levels in OA with DM were 4383.6±3075.5 μM and 5034.5±5153.9 μM, respectively, 2815.2±2224.6 μM, and 1841.8±1082.7μM for OA without DM. The difference in hexose levels between DM and non-DM patients were significant regardless of the type of samples as expected (all P<0.04). The hexose level in the SF of DM patients was higher than that in the plasma, but was not statistically significant.

**Table 1:** Descriptive statistics of the study population*.

	OA+DM (n = 46)	OA (n = 38)	*p*
Age (yrs)	68±6.6	62.7±8.8	0.01
Sex (F%)	50%	53%	1
BMI (kg/m^2^)	37.0±6.0	31.6±6.5	0.02

*F: female BMI: body mass index

The concentrations of MG, MG-H1, CML, CEL, PC ae C34:3 and PC ae C36:3 were measured in both plasma and SF samples. Because all the measurements were not normally distributed, data was log_10_ transformed for subsequent analyses.

**Fig 1:**
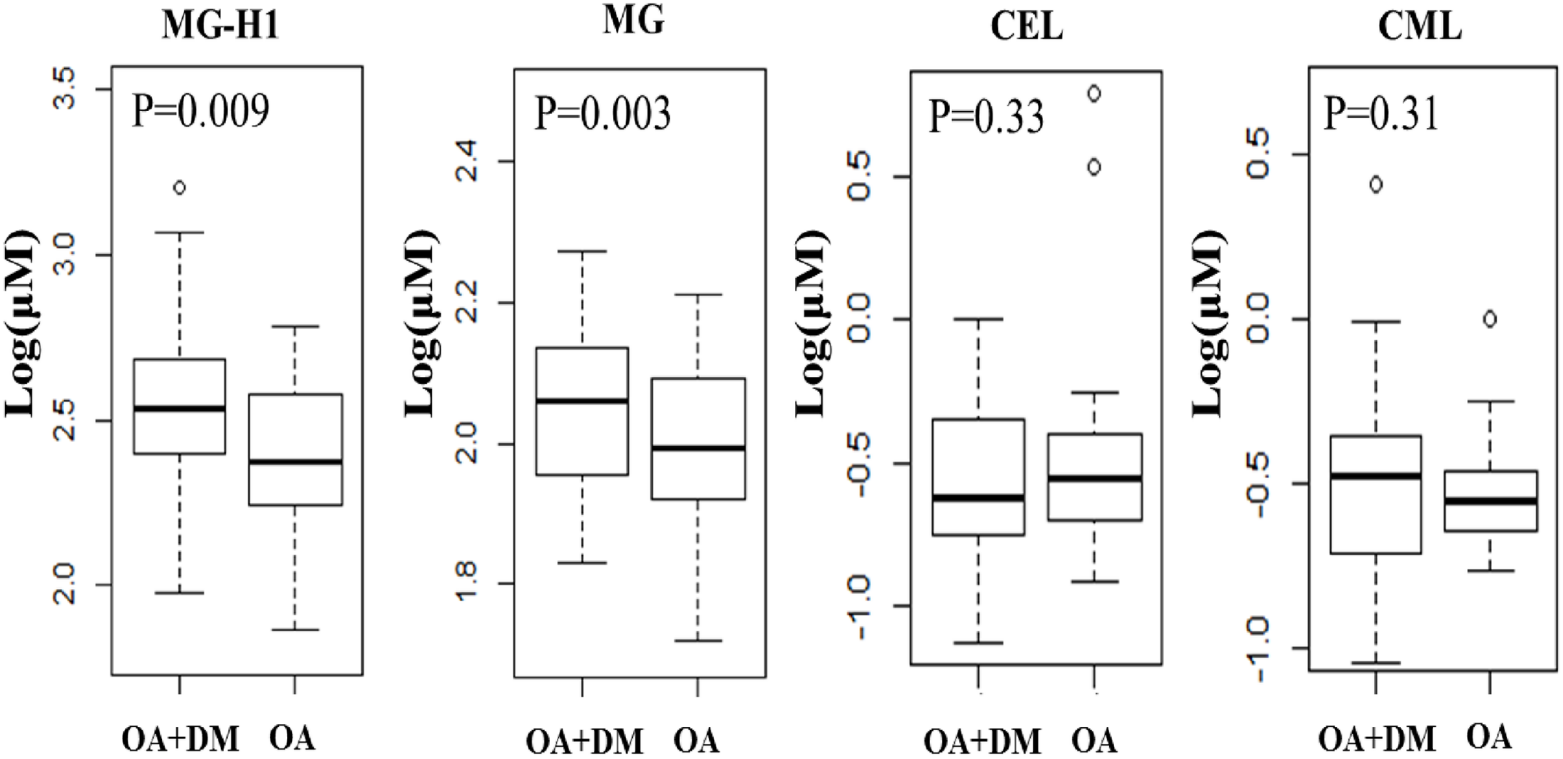
The concentrations of MG-H1, MG, CEL and CML in SF of OA and OA+DM patients. (DM: diabetes). P values were adjusted for sex, age and BMI.

None of the plasma concentrations of MG, MG-H1, CML and CEL was significantly different between OA with and without DM. However, the SF concentration of MG-H1 in OA patients with DM was 2.53±2.29 (log_10_ ng/ml), which was significantly higher than that in OA without DM patients (2.29±2.06, log_10_ ng/ml) (P = 0.01) (Fig 1). Similarly, the SF concentration of MG in OA patients with DM (2.07±1.49, log_10_ ng/ml) was also significantly higher than that in OA patients without DM (2.01±1.41, log_10_ ng/ml; P = 0.03) (Fig 1). The statistical significances of the differences were maintained after adjustment for age, BMI, sex, and hexose level (P = 0.03 for MG-H1 and P = 0.02 for MG). However, the SF concentrations of CML and CEL were not significantly different between OA with and without DM (Fig 1).

**Fig 2:**
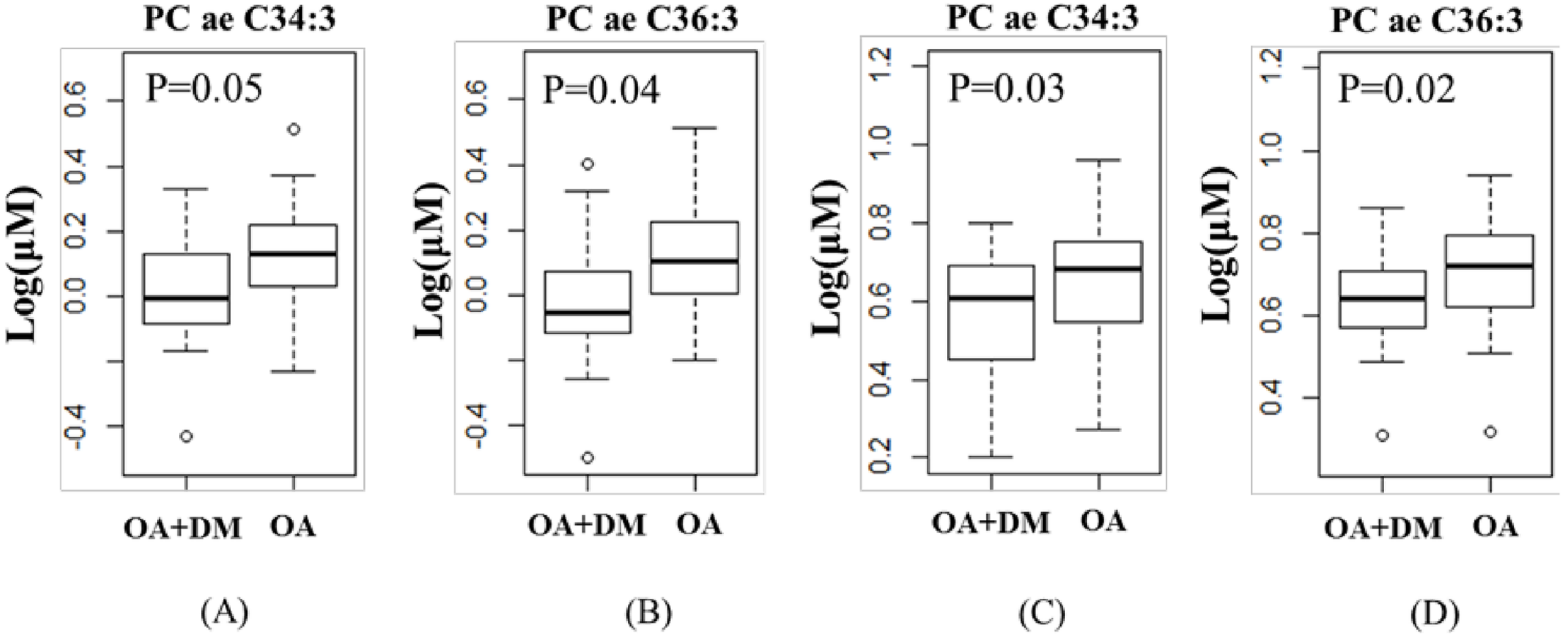
The concentrations of PC ae C34:3 and PC ae C36:3 in SF (A and B) and plasma (C and D) of OA and OA+DM patients. (DM: diabetes). P values were adjusted for sex, age and BMI.

Similar to our previous findings [7], the concentrations of PC ae C34:3 and PC ae C36:3 in SF and plasma were significant lower (all p<0.05) in OA patients with DM than that in OA patients without DM (Fig 2). For SF analysis, that the concentration of PC ae C34:3 and PC ae C36:3 in OA patients with DM were 0.015±0.18 (log_10_ ng/ml) and 0.003±0.21 (log_10_ ng/ml), respectively, which were 23% and 19% significantly lower (p = 0.04) than that in OA patients without DM whose concentrations were 0.018±0.15 (log_10_ ng/ml) and 0.010±0.15 (log_10_ ng/ml), respectively (Fig 2). The overall plasma concentrations of both metabolites were higher than that in SF (Fig 2), but similar to the SF results, OA patients with DM had a concentration reduced by 34% and 27%, respectively for PC ae C34:3 and PC ae C36:3, compared to OA without DM (all p<0.03; Fig 2). The differences remained statistically significant after adjustment of sex, age and BMI (P<0.05).

Further, we found that MG-H1concontration in SF was negatively correlated with PC ae C34:3 (ρ = -0.34, p = 0.02) and PC ae C36:3 (ρ = -0.39, p = 0.028), but none of the other three AGEs under study showed significant correlation with the two PCs. The correlation remained significant after adjustment for age, sex, BMI, hexose level (p<0.05).

## Discussion

To the best of our knowledge, this is the first study examining levels of AGE precursor MG, and AGEs including MG-H1, CML, and CEL in both plasma and SF of OA patients with and without DM. We demonstrated that increased glycation, evidenced by higher levels of MG and MG-H1 in SF, was associated with altered phosphatidylcholine metabolism, specifically certain plasmalogens within the joint. These relationships may provide insights into the underlying pathogenic process mediating the association between OA and DM observed in epidemiological studies.

MG, a potent glycation agent, is a highly reactive α-ketoaldehyde formed by both enzymatic and non-enzymatic processes using glycolytic intermediates or sugar [23]. MG-H1 and CEL result from MG-mediated damage of protein arginine and lysine residues, respectively. MG-modified proteins undergo cellular proteolysis and releasing free MG-H1 for excretion. Therefore, MG-H1 found in body fluids most likely originates from cells releasing small molecule waste into circulation for disposal. Based on this assumption, SF MG-H1 levels could reflect more closely the in situ breakdown of glycated intracellular and extracellular proteins by cells and tissues in immediate contact and surrounding the joint. MG-H1 is the major AGE in proteins of tissues and body fluids [24]. It increases in DM and is associated with vascular complications, renal failure, arthritis (OA and rheumatic arthritis) and ageing [25–28]. In the current study, SF levels of both MG and MG-H1 were higher in OA with DM than that in OA without DM, presenting evidence in support of a putative mechanism explaining the relationship between OA and DM in epidemiological studies. That is, the accumulation of MG and MG-H1 in the SF may be evidence of a role intracellular or extracellular protein glycation in the joint area, which may be higher in DM, mediating down-stream tissue damage in the local environment. Interestingly, plasma concentrations of MG and MG-H1 were not significantly different between both groups. While, the reason for this is not known, we speculate that a modifying effect of antidiabetic drugs on plasma glycemia, glycation of blood plasma proteins, and major tissues giving rise to free AGEs, like MG-H1, that eventually appear in blood for excretion may be at work. The most frequently used drugs for the treatment of DM include suppressors of hepatic gluconeogenesis (metformin) [29], insulin-sensitizing PPAR agonists (pioglitazone) [30], and insulin secretagogues (sulfonylureas) [31]. Many studies have reported the inhibiting effect of these drugs on AGE formation [32–35]. In addition, in a study involved 22 patients with PCOS and 22 healthy women, Diamanti-Kandarakis et al. [36] also found that plasma AGEs levels were reduced significantly after 6 months of metformin administration in women with polycystic ovary syndrome. Thus, we speculate that potential antidiabetic drugs may have immediate effects on reducing blood glucose level and inhibiting the production of AGEs in the blood, but the effect on reducing tissue glucose levels and non-enzymatic glycation reactions in the joint and SF may be lagging, leading to elevated production and accumulation of AGEs, particularly MG and MG-H1. While we did not measure glucose levels in the same samples used in the study, we measured hexose levels, which represent more than 90% of glucose. Indeed, SF hexose levels in OA patients with DM were higher than that in plasma and all OA with DM patients had higher hexose levels than those without in both SF and plasma, supporting this hypothesis.

In cell and animal studies, AGEs have been implicated as key pathogenic factors in initiation and progression of OA and DM [28,37]. The main mechanisms involve altered function of many intra- and extracellular proteins and generation of reactive oxygen species (ROS) by the activation of the receptor for AGEs (RAGE) [13]. Increased ROS leads to the induction of lipid peroxidation. Lipid peroxidation is considered as the main molecular mechanisms involved in the oxidative damage to cell structures and in the toxic process that leads to cell death [38]. Acyl-alkyl-phosphatidylcholines has been shown to have antioxidant properties by preventing plasma lipoprotein oxidation [39, 40]. In the present study, MG-H1 negatively correlates with the levels of PC ae C34:3 and PC ae C36:3, suggesting that decreased levels of these two polyunsaturated lipids in OA and DM patients could be due to the over lipid oxidation caused by AGEs, but also loss of an important antioxidant with potential to protect from free radical damage. Unsaturated phosphatidylcholines are the main constituent phospholipids that cover on the surface of the cartilage tissue and have an important role in lubrication [41], load-bearing function [42] and maintaining normal physiological functions of joint cartilage [43]. Reduced levels of the antioxidant potential of the PC plasminogen would have deleterious effects on initiation and progression of OA.

CML and CEL are also major protein bound AGEs found in tissue and blood and have been established as ligands of RAGE [44, 45]. We measured their plasma and SF levels in the current study, but did not find a significant difference between the OA patients with and without DM. This may be attributed to that CML and CEL in blood are mainly coming from proteins with relatively short half-life and are reflecting relatively short-term changes in glycemia and events occurring in circulation. Moreover, the SF proteome shares considerable similarity with that of plasma, no doubt because most of these proteins originate from plasma [46]. From this perspective, CML and CEL levels should reflect changes in glycation event occurring in blood plasma.

There are some limitations in the study. Firstly, the sample size was relatively small, but a post hoc power calculation suggested that we had 98.5% study power to detect the observed difference with the given sample size. Secondly, we assessed only four major AGEs in the current study, thus we may miss other AGEs that might also play a role in OA with DM. However, AGEs form by non-enzymatic processes and the major AGEs formed were covered in this study. Thirdly, patients were on various medications that have a potential modifying effect on inflammatory processes and glycemic conditions that may profoundly modify the AGE response. While these effects can be significant these treatments do not normalize glycemia, but may require larger patient populations and greater powered studies to reveal differences that may be present. It is worth noting that formation of AGE precursors like MG and AGEs involve mechanisms more complex than just glycemia alone, and the effects of drugs like metformin on AGE levels involve interactions that are independent of direct effects of the drug on glycemia [47]. Fourthly, Diabetes could cause many complications that may have indirect effects on phosphatidylcholine metabolism. However, very few subjects had diabetic complications including cardiovascular diseases (CVD), renal damage, and eye damage. Thus we did not have sufficient power to examine the confounding effects of these diabetic complications on the observed association. Fifthly, the results would be more conclusive if we had negative controls. However, obtaining SF samples from healthy subjects are nearly impossible ethically. However, obtaining SF samples from healthy subjects are nearly impossible ethically. And lastly, the study was cross-sectional; a longitudinal study is required to establish a causal relationship for the observed association in the current study.

In summary, we demonstrated that both MG-H1 and MG concentrations in SF were elevated in OA patients with DM and associated with the levels of PC ae C34:3 and PC ae C36:3. These findings might assist to establish a relationship between AGE mediated tissue damage especially in the joint, and derangement in phosphotidylcholine metabolism particularly affecting PC plasmalogens. These relationships might help form the mechanistic relationship explaining the epidemiological association between OA and DM.
